# Comorbidities associated with severe asthma

**DOI:** 10.2500/jprm.2019.190006

**Published:** 2019-12

**Authors:** Gayatri B. Patel, Anju T. Peters

**Affiliations:** 1 Division of Allergy and Immunology, Department of Medicine, Feinberg School of Medicine, Northwestern University, Chicago, Illinois

## Abstract

**Background:**

Severe asthma can be a challenging disease to manage by the provider and by the patient, supported by evidence of increased health-care utilization by this population. Patients with severe asthma should be screened for comorbidities because these often contribute to poorly controlled asthma. The impact of comorbidities, however, are not completely understood.

**Objective:**

To review common comorbidities and their impact on severe asthma.

**Methods:**

A review of relevant clinical research studies that examined comorbidities in severe or difficult-to-treat asthma.

**Results:**

A number of comorbid diseases, including rhinitis, rhinosinusitis, gastroesophageal reflux, and obstructive sleep apnea, are associated with severe or difficult-to-treat asthma. If present and untreated, these conditions may adversely affect asthma control, quality of life, and/or lung function, despite adequate treatment with step-up asthma controller therapy.

**Conclusion:**

Treatable comorbidities are associated with severe and difficult-to-control asthma. Failure to recognize these comorbidities may divert appropriate care and increase disease burden. Assessment and management of these risk factors may contribute to improved asthma outcome; however, more investigation is needed to understand the relationship of comorbidities and asthma due to inconsistency in the findings.

Severe asthma is defined as asthma that requires treatment with high-dose inhaled corticosteroids (ICS) plus a second controller or systemic corticosteroids needed for 50% of the previous year to prevent asthma from becoming uncontrolled or which remains uncontrolled despite therapy.^1^ This definition requires confirmation of the correct diagnosis, good treatment adherence, and correct inhaler technique. The estimated prevalence of severe asthma is 5–10% of the total asthma population, and an estimated 3.6% have severe refractory asthma.^2^ Although severe asthma accounts for a small fraction of all patients with asthma, this subset is responsible for a disproportionate amount of health-care utilization and economic burden due to increased exacerbations, the higher level of medications dispensed, and asthma-related morbidities.^3^ Physician evaluation of factors, *e.g*., comorbid conditions, that contribute to the control of severe asthma is important to reducing this burden.

Comorbidities are often associated with severe asthma.^1^ In an observational study of 2132 patients who were identified from a Dutch pharmacy data base, 58% were considered as having difficult-to-treat asthma.^4^ Of this group, 92% had at least one comorbidity. Furthermore, this group, overall, had more comorbidities compared with those without difficult-to-control asthma. This is relevant because comorbid diseases in severe asthma are known risk factors for poor asthma control, recurrent exacerbations, and diminished quality of life.^5^ Recognition, followed by modification or treatment of comorbidities in these patients may reduce the overall disease burden.^6^ In this review, we discussed the impact of comorbid rhinitis, rhinosinusitis, gastroesophageal reflux (GER), and obstructive sleep apnea (OSA) on severe persistent or difficult-to-control asthma. Other comorbid conditions, such as obesity, dysfunctional breathing, vocal cord dysfunction, and anxiety and/or depression, were identified but not discussed in this review (Fig. 1).

## RHINITIS

Rhinitis and asthma are frequently coexisting diseases and are often referred to as united airways disease.^7^ The prevalence of nasal symptoms range from 6% to 85% in patients with asthma.^8^ A European asthma cohort, the Unbiased Biomarkers in Prediction of Respiratory Disease Outcomes (U-BIOPRED) consortium, performed a prospective study of 610 adults with severe asthma to better characterize baseline clinical characteristics of these patients.^9^ Of the 311 patients with severe nonsmoking asthma, 59.2% had diagnosed allergic rhinitis (AR) and 14.8% had diagnosed nonallergic rhinitis (NAR). The impact of both AR and NAR on asthma has been studied. Singh *et al.*^10^ showed, in a retrospective cohort study, that patients hospitalized with a primary diagnosis of asthma and with a history of AR or NAR had a 30-day readmission adjusted hazard ratio of 4.4 and 3.7, respectively, compared with those without rhinitis. This striking finding of rhinitis as a predictive factor for readmission is only an association because causality cannot be determined from this study. One hypothesis, however, is that uncontrolled rhinitis may have triggered an asthma exacerbation, although this was not ascertained from the chart. Further studies that examine how treatment of uncontrolled chronic rhinitis influences readmission rates will provide more insight into the interplay between rhinitis and asthma.

Another example of how environmental aeroallergen sensitization has an impact on asthma is demonstrated by the phenomena of thunderstorm asthma. This is a well-described occurrence with the largest outbreak taking place on Monday, November 21, 2016, in Melbourne, Australia, that resulted in nine deaths.^11^ More than 8500 individuals presented to the emergency department for asthma symptoms in a 24-hour period during a thunderstorm. The implicated cause was rain and wind gust combined with extreme levels of airborne rye grass pollen, which was distributed across the city. Specifically, storms were suggested to cause the release of respirable allergenic components from the ruptured pollen grains, which can lead to an exacerbation in individuals sensitized to asthma. Hew *et al.*^12^ contacted 1435 patients who were affected and performed chart reviews and telephone interviews, with the aim of understanding risk factors and individual susceptibilities for the hospital admission. Of these patients, 87% reported having seasonal allergic rhinitis, of whom 72% reported moderate or severe symptoms (based on established criteria).^12^ The relationship of thunderstorm asthma among those with grass allergy underscores the association between allergic rhinitis and asthma exacerbations.

The use of intranasal corticosteroid sprays (INCS) for treatment of rhinitis and its impact on asthma has been studied; however, a systematic review with meta-analysis that evaluated the role of INCS in patients with AR on daily ICS for asthma did not show any significant improvements.^13^ The majority of the trials, however, had excluded patients with severe asthma, which is arguably the group that would see potential improvement. In summary, the benefit of INCS on patients with severe asthma still needs to be further examined. Physicians should screen for uncontrolled rhinitis and optimize control based on Allergic Rhinitis and Influence on Asthma guidelines^8^ recommendations in patients with or without asthma.

## RHINOSINUSITIS

Chronic rhinosinusitis (CRS) is closely associated with allergic rhinitis and asthma.^14^ A study that used the Severe Asthma Research Program (SARP) cohort in the United States assessed phenotypic characterization of asthma in 726 subjects.^15^ There was a 45% prevalence of comorbid sinus disease with increased prevalence in older individuals with more severe asthma. In Europe, a cross-sectional study of 136 patients with severe asthma found that severe sinonasal disease (confirmed by nasal endoscopy and computed tomography of the sinus) was associated with frequent asthma exacerbations.^16^ A larger U.S. study, of 703 patients in Severe Asthma Research Program - 3 (SARP-3), also confirmed that sinusitis was associated with more frequent asthma exacerbations.^17^ Ek *et al.*^18^ showed, in a study with 605 patients with asthma, that, in addition to being associated with frequent asthma exacerbations, comorbid CRS was associated with worse quality of life compared with asthma alone. Furthermore, CRS and coexisting asthma had decreased forced expiratory volume in the first second of expiration FEV_1_% predicted (88.4%) compared with patients with asthma alone (91.9%) (*p* < 0.05).

There is limited literature of patients with CRS and asthma that surgical intervention may improve asthma symptoms and lung function.^19,20^ A systematic review and meta-analysis that included three randomized control trials and 10 case series showed low-quality evidence to support the association of functional endoscopic surgery in CRS with lung function improvement.^21^ The authors of this review recommend shared decision-making with the patient and the physician on the risk to benefits ratio and expectations of pursuing endoscopic surgery because benefit on asthma improvement is not completely understood.

## GER

The estimated prevalence of GER in asthma is 33–90%, depending on the definition of GER and measures used to obtain this diagnosis.^22–24^ In a systematic review, it was estimated that 59% of adults with asthma have reflux symptoms or 51% of adults when using pH monitoring.^25^ In a longitudinal, observational 10-year follow-up study, The Epidemiology and Natural History of Asthma: Outcomes and Treatment Regimens II, which included 341 subjects with physician-assessed severe or difficult-to-treat asthma, 46.3% self-reported GER disease.^26^ Analysis of the results of these studies indicated that approximately 50% of patients with asthma have symptomatic reflux. GER seems to increase in prevalence in those with increased numbers of exacerbations, which was shown in the both the SARP-3^17^ and U-BIOPRED^9^ cohort studies, mentioned earlier in this review. A study of patients with difficult-to-treat asthma noted an odds ratio of 4.9 for association of GER and frequent asthma exacerbations.^16^

Several well-designed clinical trials examined whether proton-pump inhibitors improve asthma control in adult patients with asthma and either asymptomatic or symptomatic reflux.^27–30^ A double-blind, placebo-controlled, randomized clinical trial of esomeprazole in patients with inadequately controlled asthma and with minimum or no GER symptoms found no significant difference in asthma control, lung function, or quality of life compared with placebo.^30^ GER was confirmed by pH monitoring in 40% of the patients in the esomeprazole group. A study limitation was that subjects with FEV_1_ < 50% of their predicted value were excluded. Similarly, in children with poorly controlled asthma on ICS without symptomatic GER, lansoprazole did not have any significant benefit on asthma control, lung function, or quality of life compared with placebo.^29^ Analysis of these data indicated that patients with poorly controlled asthma and with asymptomatic GER will not likely benefit from proton-pump inhibitor treatment.

Recommendations for treatment of GER in patients who are symptomatic with severe asthma is less clear. Kiljander *et al.*^28^ conducted a large multicenter, double-blind, placebo controlled trial with 961 patients with moderate-to-severe persistent asthma and acid reflux symptoms, and randomized to either esomeprazole once daily, esomeprazole twice daily, or placebo. Although there was a statistically significant improvement in lung function with esomeprazole compared with placebo, this was not considered clinically significant (FEV_1_ of 0.09 L [*p* = 0.0039] versus FEV_1_ of 0.12 L [*p* < .0001], for once daily dosing and twice daily dosing, respectively). Mean peak flow did improve in both treatment groups compared with placebo but did not reach statistical significance. Asthma-related quality of life was significantly improved in both treatment groups compared with placebo. There were no differences in asthma exacerbation rates. In comparison, an older study, from 2005, conducted by Littner *et al.*,^27^ with a similar design, showed a significant reduction in asthma exacerbations in those taking lansoprazole compared with placebo (8.1% versus 20.4%, *p* = 0.017) and improvement in the asthma quality-of-life questionnaire with standardized activities (AQLQS) emotional function domain in those who took lansoprazole (*p* = 0.025). There were no changes in lung function. Overall, analysis of the data in favor or against treatment with proton-pump inhibitor in patients symptomatic for GER with severe asthma are not consistent.

Based on the available data, there is adequate support to recommend a trial of proton-pump inhibitor once or twice daily for a minimum of 4–6 weeks if the patient has asthma and symptomatic reflux. If GER symptoms are not responsive to treatment, then a 24-hour pH test can be done to further understand the etiology of symptoms. There seems to be no benefit of treatment with a proton-pump inhibitor in patients with poorly controlled asthma and minimum or no symptoms of GER.

## OSA

Asthma with coexisting OSA is being increasingly recognized. Existing asthma can increase the risk of developing OSA, and, inversely, OSA can be a significant risk factor for asthma exacerbation.^31,32^ In a cross-sectional study of 146 patients with asthma and 157 controls, the prevalence data showed that 19.2% of the patients with asthma had OSA compared with 9.6% of the controls, and the apnea-hypopnea index (AHI) positively correlated with asthma exacerbations.^33^ A limitation of this study was that it excluded patients with FEV_1_ < 50%. In a longitudinal study that included self-reported physician diagnosed asthma of all severity types, the patients with asthma had a 27% increase in developing new-onset OSA compared with 17% in those without asthma.^31^ Furthermore, patients with asthma and untreated or unidentified high-risk OSA based on an OSA symptom questionnaire had an adjusted odds ratio of 2.87 for risk of frequent exacerbations.^32^ Other studies have documented that the OSA prevalence is greater with progressive severity of asthma.^31,34^ Julien *et al.*^34^ showed the prevalence of OSA is 88% in severe asthma, 58% in moderate asthma, and 31% in controls without asthma based on criteria that used AHI ≥ 15 events/hr.^31^

There are studies that show a beneficial role of continuous positive airway pressure (CPAP) therapy for asthma control and quality of life in patients with concomitant OSA and asthma, particularly those with severe or difficult-to-control asthma. In a prospective study that followed up 99 adult asthma patients on CPAP for 6 months for OSA, uncontrolled asthma went from 41.4% to 17.2%.^35^ The Asthma Control Questionnaire score had a statistically significant reduction from baseline to 6 months in those with moderate-to-severe persistent asthma (1.58 ± 0.95 to 1.11 ± 0.85; *p* = 0.003) and in those with increased OSA severity based on respiratory disturbance index > 30 (1.36 ± 0.92 to 0.99 ± 0.76; *p* = 0.012). Asthma-related quality of life also improved significantly in those with moderate-to-severe persistent asthma (4.87 ± 1.45 to 5.48 ± 1.24; *p* = 0.012). Interestingly, other comorbidities of asthma, including heartburn and mild rhinitis, had statistically significant improvement.

Wang *et al.*^36^ showed that patients with asthma and with OSA had a greater decline in FEV_1_ compared with those without OSA. Furthermore, the results showed that AHI severity had a moderate correlation with decline in the FEV_1_% predicted. In the severe OSA group with good adherence to CPAP (38%), there was approximately 20 mL per year less decline in FEV_1_ (mean ± SEM:41.2 ± 36.1 mL versus 69.4 ± 66.4 mL; *p* = 0.028) after treatment for 2 years. Limitations to this analysis were the small sample and the lack of a control group. Improvements in lung function have not been consistently observed among several studies. In a meta-analysis that included 12 prospective studies with a mean duration of 19.5 weeks of CPAP therapy, there was significant improvement in asthma-related quality of life in those with poorly controlled asthma but no change in FEV_1_ before and after CPAP.^37^ In summary, the use of CPAP in patients with OSA and asthma can be beneficial for asthma control and quality of life. There are insufficient data regarding the role of CPAP in lung function improvement. This can be challenging to assess because patients with asthma have variable lung function and some have fixed airflow obstruction.

## CONCLUSION

Screening and potential treatment for comorbidities of severe asthma can have positive implications on the outcome of care. Tay *et al.*^38^ showed, in a longitudinal observational study, that a structured approach to specialist care with targeted comorbidity interventions for those with confirmed difficult-to-control asthma was associated with enhanced asthma outcomes. The same group also showed that the use of validated screening questionnaires can heighten detection of comorbidities by physicians.^39^ This type of approach can help physicians effectively and efficiently prioritize assessment of comorbidities in severe asthma. Asthma subspecialist awareness of increased risk of comorbid conditions, including (but not limited to) rhinitis, sinusitis, GER, and OSA, is essential to the overall assessment in any patient with difficult-to-control or severe persistent asthma.

## Figures and Tables

**Figure 1.**
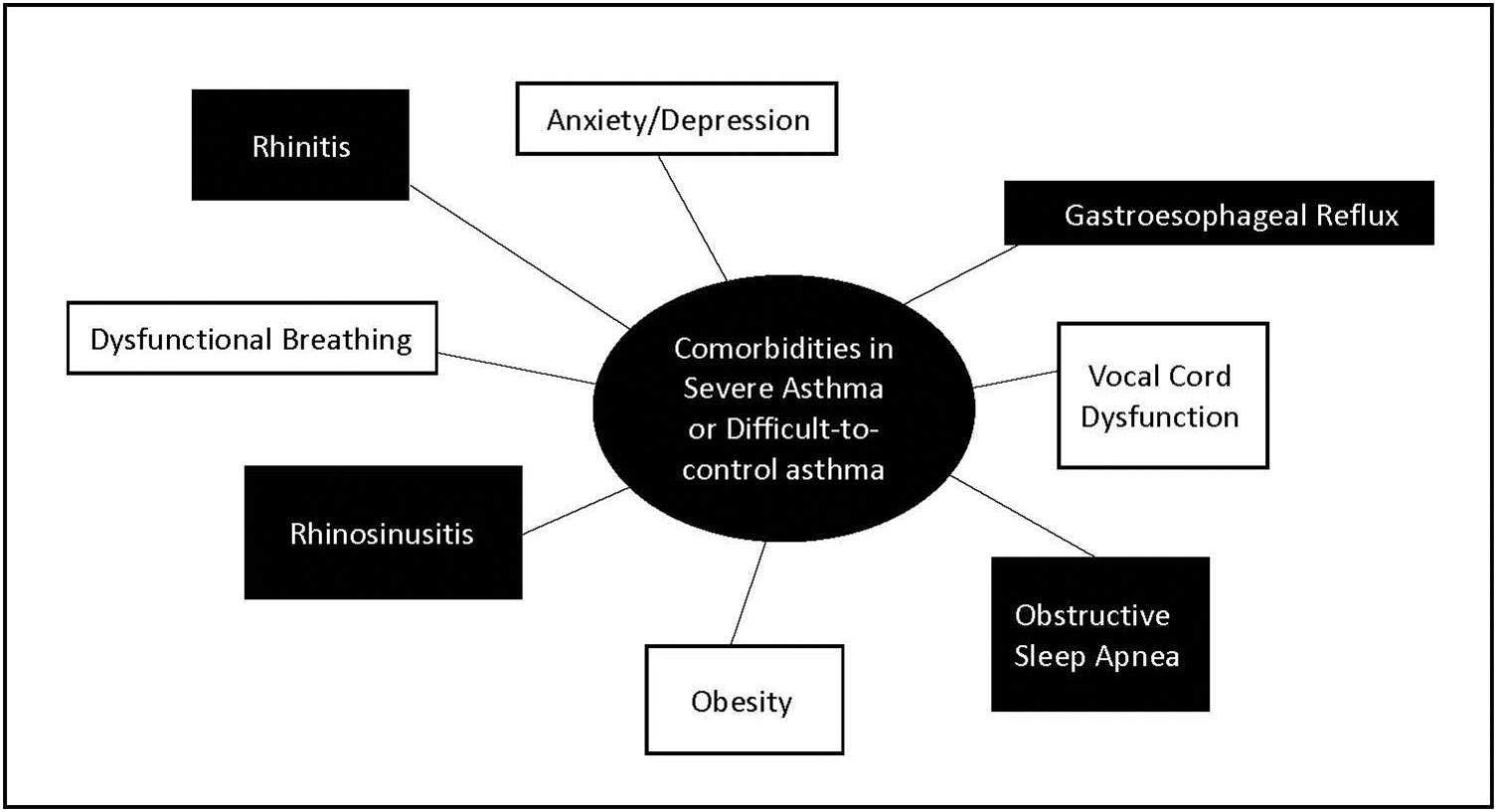
Comorbidities associated with severe asthma or difficult-to-control asthma. Comorbidities listed in the black boxes are discussed in this review.
